# Minimally Invasive Pedicle Screw Fixation Combined with Percutaneous Kyphoplasty Under O‐Arm Navigation for the Treatment of Metastatic Spinal Tumors with Posterior Wall Destruction

**DOI:** 10.1111/os.12712

**Published:** 2020-06-23

**Authors:** Zhang‐zhe Zhou, Yi‐meng Wang, Xiao Liang, Xiao Ze, Hao Liu, Kang‐wu Chen, Xiao‐yu Zhu, Zhi‐yong Sun, Zhong‐lai Qian

**Affiliations:** ^1^ Department of Orthopaedic Surgery The First Affiliated Hospital of Soochow University Suzhou China; ^2^ Department of Orthopaedic Surgery The Second Affiliated Hospital of Soochow University Suzhou China

**Keywords:** Kyphoplasty, Metastatic spinal tumors with posterior wall destruction, Minimal invasive, O‐arm navigation, Pedicle screw fixation

## Abstract

**Objective:**

To evaluate the safety and efficacy of O‐arm‐guided minimally invasive pedicle screw fixation combined with percutaneous kyphoplasty for metastatic spinal tumors with posterior wall destruction.

**Methods:**

Patients who underwent minimally invasive pedicle screw fixation combined with percutaneous kyphoplasty for pathological vertebral fractures with posterior wall defects from January 2015 to December 2017 were followed up for 1 year. Visual analogue scale (VAS), SF‐36 scores, middle vertebral height, posterior vertebral height, and the accuracy of pedicle screws were assessed preoperatively, postoperatively, and 1 year after surgery. The operation time, time from operation to discharge, blood loss, volume of bone cement, and leakage of bone cement were recorded.

**Results:**

Twenty‐three patients (13 females and 10 males) who met our criteria were followed up for 1 year. The operation time of these patients was 162.61 ± 33.47 min, the amount of bleeding was 230.87 ± 93.76 mL, the time from operation to discharge was 4.35 ± 2.42 days, and the volume of bone cement was 3.67 ± 0.63 mL. The VAS score decreased from 7.04 ± 1.07 to 2.65 ± 0.93 before surgery (*P* = 0.000) and remained at 2.57 ± 0.79 1 year after surgery. Compared with the preoperative SF‐36 scores for physical pain, physiological function, energy, and social function, the postoperative scores were significantly improved (*P* = 0.000). The height of the middle vertebral body increased from 14.47 ± 2.96 mm before surgery to 20.18 ± 2.94 mm (*P* = 0.000), and remained at 20.44 to 3.01 mm 1 year after surgery. The height of the posterior vertebral body increased from 16.56 ± 3.07 mm before operation to 22.79 ± 4.00 mm (*P* = 0.000), and 22.45 ± 3.88 mm 1 year after surgery. The 23 patients had a total of 92 pedicle screws; 85 screws were Grade A and 7 screws were Grade B. There was no leakage of bone cement after surgery.

**Conclusion:**

In the short term, O‐arm‐guided minimally invasive pedicle screw fixation combined with kyphoplasty is safe and effective in the treatment of metastatic spinal tumors with posterior wall destruction.

## Background

With the developments of the social economy and medical technology, the survival time of cancer patients is significantly prolonged^1^. The spine is the most common bone metastasis site in cancer patients^2^. In the United States, more than one million cancer patients suffer from spinal metastasis each year, of which approximately 70% are in the thoracic vertebrae, 20% are lumbar vertebrae, and 10% are cervical vertebrae^3^, ^4^. Tumors transferred to the spine can cause severe back pain, pathological vertebral fractures, and spinal cord compression symptoms^5^. Therefore, the further development of treatments options for metastatic vertebral tumors is critical.

The quality of life of patients is significantly reduced^6^. Analgesics, radiotherapy, chemotherapy, and other conservative drugs are sometimes ineffective or short‐lived^7^. For advanced cancer patients, traditional open surgery is usually not undertaken because of their short life expectancy and poor tolerance^8^. Minimally invasive surgery for metastatic vertebral tumors appears to be a perfect solution, which can not only be used for palliative treatment but also reduce surgical complications and accelerate postoperative recovery^9^. Minimally invasive pedicle screw fixation is not only associated with less injury and faster recovery after surgery but also does not require dissection of paravertebral muscles in a large area, which is undertaken to avoid complications such as muscle injury^10^. The traditional fluoroscopy operation with a C‐arm machine is challenging. When limited to fluoroscopy, the accuracy of pedicle screw placement is restricted, and damage to blood vessels and nerves can occur. The O‐arm navigation system can greatly reduce the risk of intraoperative nerve injury and shorten the operation time. Kyphoplasty requires a pneumatic balloon to restore vertebral height to reduce cement injection pressure and the risk of vertebral leakage^11^. Tumor tissue invades the posterior column, causing spinal destruction and instability^12^. However, vertebral wall destruction is a known risk factor for cement leakage, and the posterior wall defect may increase the risk of cement leakage and compression of the spinal cord or nerve root. The posterior wall defect is a relative but not an absolute contraindication of kyphoplasty^13^, ^14^. However, there are no clear guidelines on this issue, and the practice of spine centers varies.

In this study, we retrospectively followed up the patients who received the treatment of minimally invasive pedicle screw fixation combined with percutaneous kyphoplasty under O‐arm navigation for metastatic spinal tumors with posterior wall destruction. The major outcomes were evaluated: (i) to evaluate the accuracy of pedicle screw under O‐arm navigation; (ii) to observe the operative time, intraoperative blood loss and other operative indexes; and (iii) to assess the therapeutic effect.

## Materials and Methods

**Figure 1:**
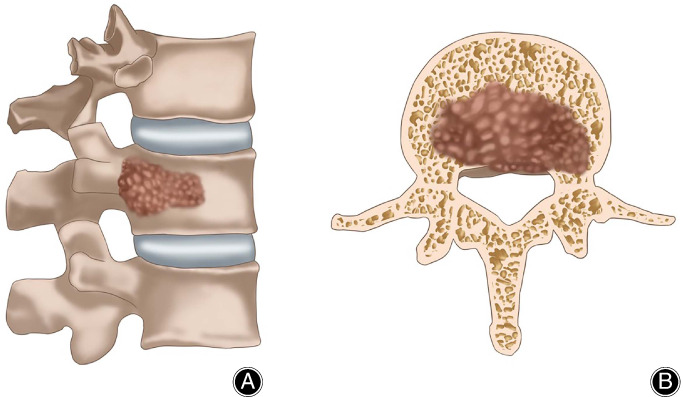
(A) The tumor tissue invaded and destroyed the vertebrae, but the upper and lower endplates and anterior walls of the vertebrae were intact. (B) The tumor tissue invaded the vertebral body and involved the posterior wall but did not invade the vertebral canal to compress the spinal cord.

This study was approved by the Ethics Committee of the First Affiliated Hospital of Soochow University. A total of 27 patients who underwent minimally invasive pedicle screw fixation combined with percutaneous kyphoplasty for pathological vertebral fractures with posterior wall defects from January 2015 to December 2017 met our inclusion and exclusion criteria. As shown in Fig. 1, all patients’ metastatic vertebral tumors invaded the posterior wall of the vertebral body, but the tumor tissue did not invade the vertebral canal to compress the spinal cord. Inclusion criteria followed the PICOS principle: (i) Participant (metastatic vertebral tumors, diagnosed by a multidisciplinary team, which included an experienced radiologist, orthopaedic surgeon and oncologist); (ii) Intervention (minimally invasive pedicle screw fixation combined with percutaneous kyphoplasty under O‐arm navigation); (iii) Comparison (comparison of preoperative and postoperative status); (iv) Outcome (minimally invasive pedicle screw fixation combined with percutaneous kyphoplasty under O‐arm navigation for the treatment of metastatic spinal tumors with posterior wall destruction is feasible and effective); and (v) Study design (retrospective study). Exclusion criteria were: (i) infections, psychiatric disorders, coagulation disorders and severe diseases, such as cardiovascular and cerebrovascular diseases; (ii) patients diagnosed with primary malignant vertebral tumors; (iii) patients with compression in the spinal cord or nerve roots; (iv) patients lost to follow up; and (v) patients with expected survival less than 1 year.

### *Surgical Operation*

#### *Anesthesia and Position*

**Figure 2:**
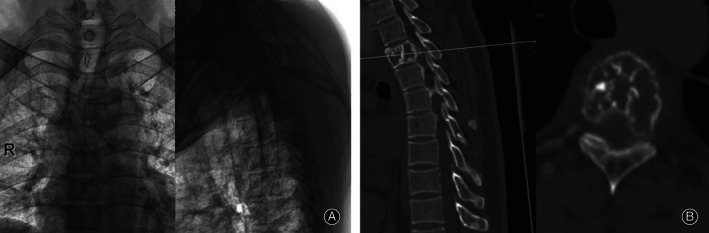
The patient had unbearable back pain and came to the hospital for treatment: (A) X‐ray showed no collapse of the T_4_ vertebral body shape and (B) the CT showed severe bone destruction.

**Figure 3:**
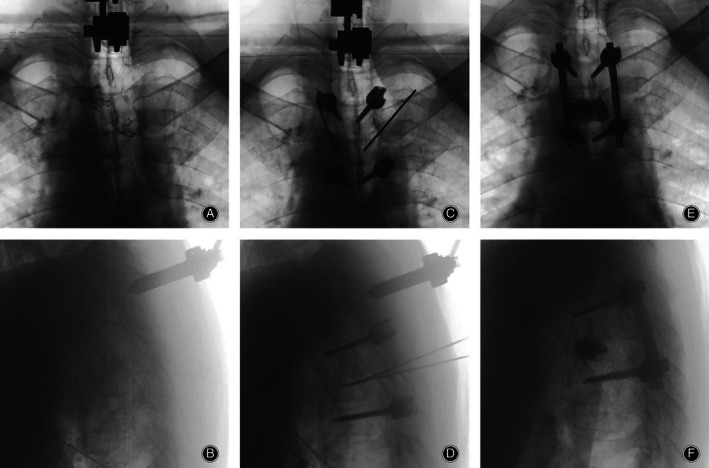
(A, B) Fluoroscopy of the O‐arm machine before placing screws. (C, D) Percutaneous placement of four pedicle screws *via* O‐arm machine at T_3_ and T_5_; the screws penetrate the pedicle into the vertebral body accurately. (E, F) Polymethylmethacrylate (PMMA) was injected into the T_4_ diseased vertebral body, and no leakage of PMMA was observed after surgery.

Take the operation of a T_4_ tumor patient as an example (Fig. 2). After the patient provided written informed consent, the surgery was performed by two senior professional spine surgeons in our hospital under general anesthesia, with the patient placed in the extended prone position, with padding beneath the upper chest and pelvic regions, on a carbon fiber table (Fig. 3A and B).

#### *Exposure and Placement*

Percutaneous pedicle screws were placed into segments adjacent to metastatic vertebrae through the posterior minimal approach guided by the O‐arm navigation system (Fig. 2C and D).

#### *Kyphoplasty*

The whole kyphoplasty was performed after the placement of the pedicle screws. A bioptic examination of the affected vertebrae was conducted prior to injecting the cement. This allowed the operator to obtain a small cylindrical sample of tissue and the polymethylmethacrylate was used as filler was used as filler.

#### *Reconstruction*

After the kyphoplasty, the sextant rods were pre‐contoured into a curvilinear shape that precisely matched the contour of the rod inserter. The rods were then placed in a standard submuscular position with minimal manipulation and no muscle dissection (Fig. 2E and F).

#### *Monitoring and Recording*

After the operations, the patients were monitored for 6 hours. The operative time, fluoroscopy time, and cement volume were registered during the operation. Cement leakage was recorded postoperatively.

### *Clinical and Radiographic evaluation*

#### *Vertebral Height*

A retrospective analysis was made based on case data and outpatient review results. Lateral X‐ray films were used to measure the middle vertebral height (MVH) and the posterior vertebral height (PVH) preoperatively, postoperatively, and 1 year postoperatively. MVH represented vertical height at the intersection of the sagittal and coronal planes in the center of the vertebral body. PVH represented vertical height at posterior margin of the sagittal plane of the vertebral body.

#### *Visual Analogue Scale*

The visual analogue scale (VAS) was used to evaluate the degree of back pain, where the pain increase is reflected in a numerical value from 0 to 10: 0 means no pain and 10 means the most severe and unbearable pain.

#### *SF‐36*

The SF‐36 questionnaire is a comprehensive health‐related quality of life assessment tool consisting of 36 questions. Bodily pain (BP), vitality (VT), physical function (PF), and social function (SF) have high reliability and responsiveness in patients with spinal injuries. The higher the score, the better the health status.

#### *Pedicle Screw Position*

To understand the position of the pedicle screw under the O‐arm navigation, the accuracy of pedicle screw position was evaluated by Gertzbein and Robbins scales: Grade A, excellent screw position without cortical perforation; Grade B, pedicle cortical breach <2 mm; Grade C, 2 mm ≤ pedicle cortical breach <4 mm; Grade D, 4 mm ≤ pedicle cortical breach <6 mm; Grade E, pedicle cortical breach ≥6 mm^14^.

### *Statistical Analysis*

SPSS software (SPSS 23.0, USA) was used to evaluate and analyze the mean and standard deviation of posterior vertebral height change, SF‐36 scores, and VAS scores. Paired *t*‐tests were used to compare outcomes at different time points. If the *P*‐value was less than 0.05, the difference was considered statistically significant.

## Results

**Figure 4:**
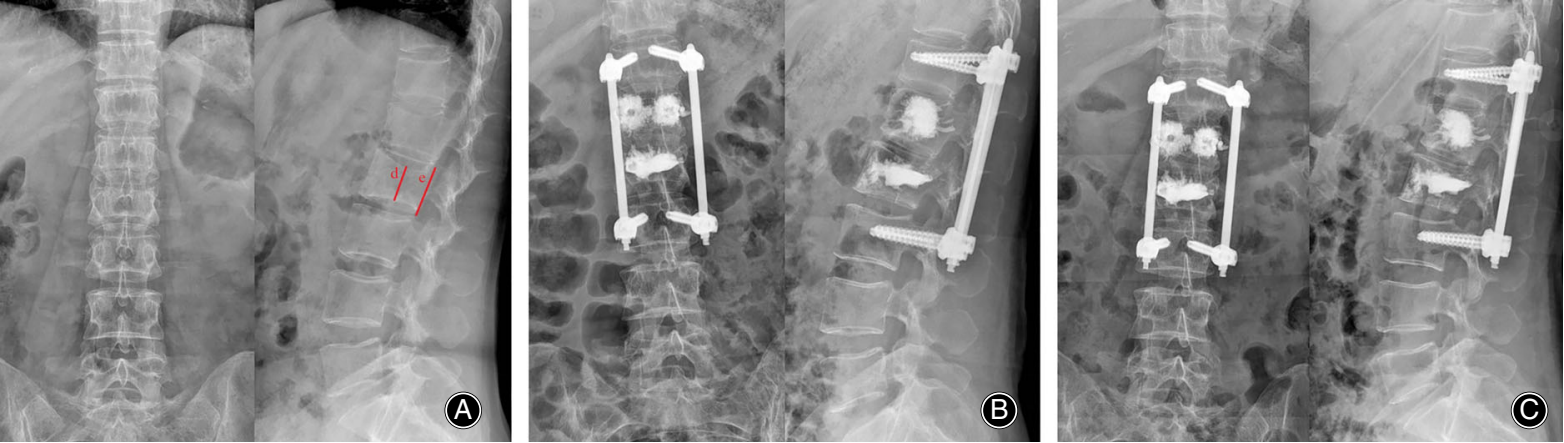
Preoperative and postoperative X‐ray of the illustrative case A. (A) Preoperative X‐ray demonstrated that L_1_ and L_2_ vertebral morphology changed; (B) postoperative X‐ray demonstrated that L_1_ and L_2_ vertebral body were filled with bone cement and the spinal stability was restored by pedicle fixation; (C) 1 year after operation, X‐ray demonstrated that the shape of the vertebral body remained intact; (D) line d represents the schematic diagram of middle vertebral height; and (E) line e represents the schematic diagram of the posterior vertebral height.

**Figure 5:**
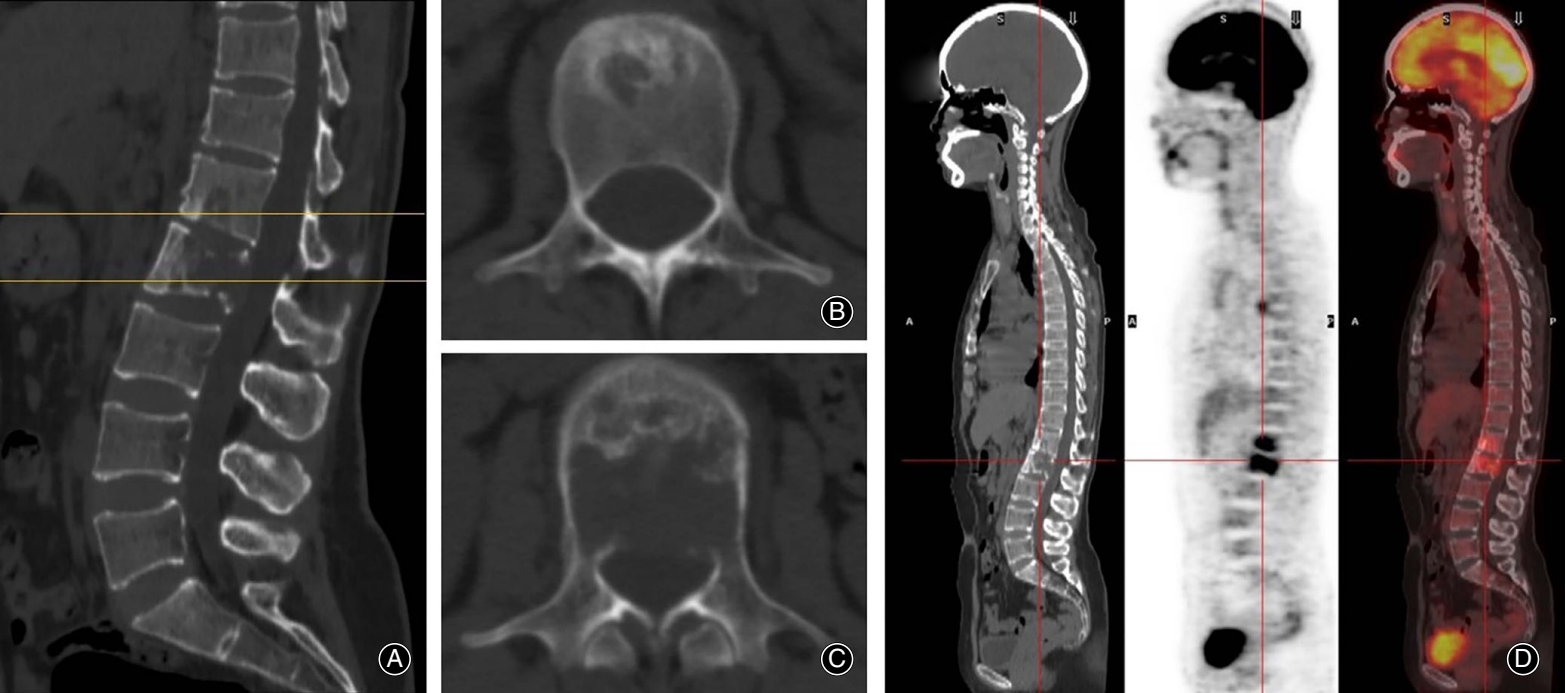
Preoperative CT and PET‐CT of illustrative case A. (A, B, C) Preoperative CT showed bone destruction at L_1_ and the destruction of posterior vertebral wall at L_1_ and L_2_; and (D) preoperative PET‐CT showed that the uptake rate of tracers at L_1_ and L_2_ sites was higher than that at other sites.

**Figure 6:**
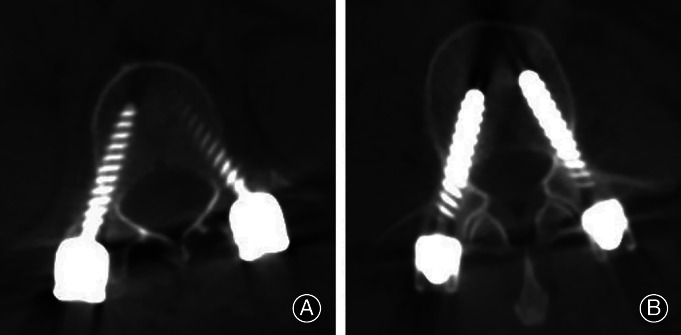
Postoperative CT of the illustrative case A. (A, B) Postoperative CT showed the accuracy of the pedicle screw position at T_12_ and L_3_; the accuracies of pedicle screw positions were grade A.

**Figure 7:**
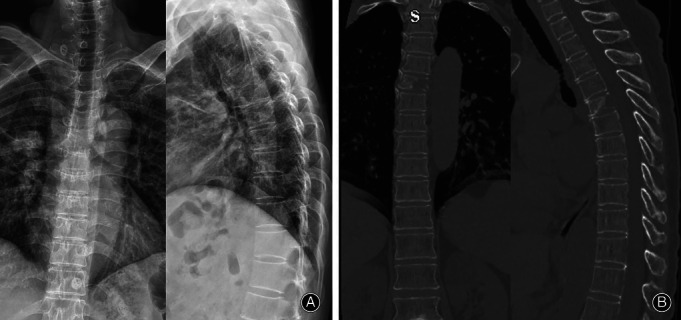
Preoperative X‐ray and CT of the illustrative case B. (A, B) Preoperative X‐ray and CT showed that the shape of the T_5_ vertebral body changes and the destruction of the vertebral body bone involve the posterior wall damage.

**Figure 8:**
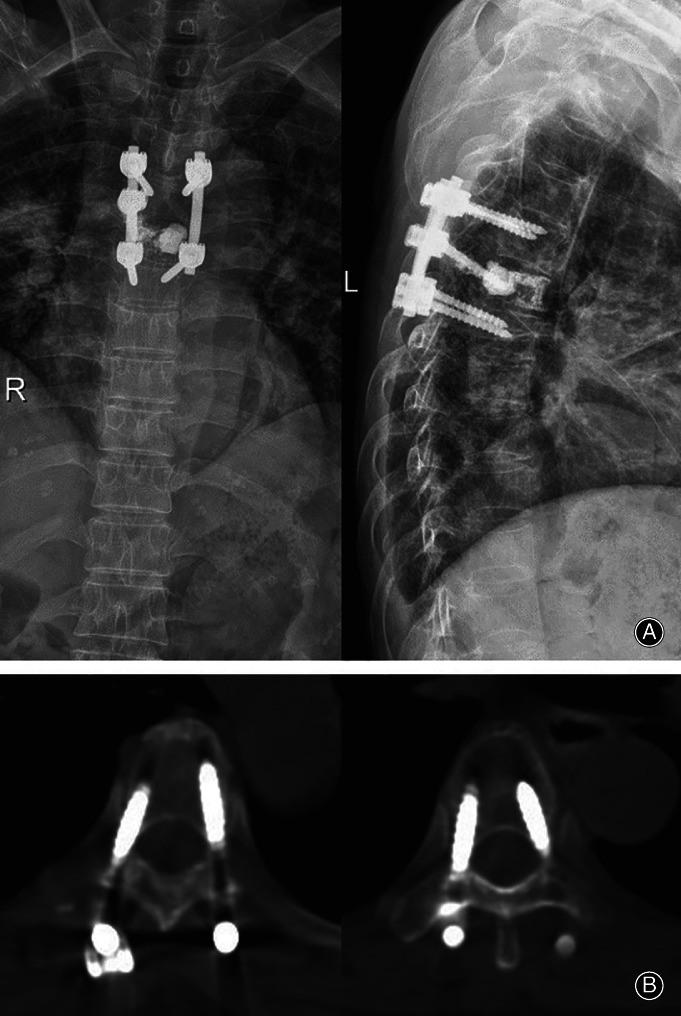
Postoperative X‐ray and CT of illustrative case B. (A) Postoperative X‐ray showed that the T_5_ vertebral body was filled with bone cement and the spinal stability was restored by pedicle fixation. (B) Postoperative CT showed the accuracy of the pedicle screw position at T_4_ and T_6_; the accuracies of pedicle screw positions were grade A.

We performed a 1‐year follow up for 23/27 patients. As a result of underlying disease, 2 patients died within 1 year and 2 patients were lost to follow up. In this study, 23 patients (13 females and 10 males) who met the inclusion and exclusion criteria were followed up, including 9 patients with breast cancer, 7 patients with lung cancer, 2 patients with liver cancer, 2 patients with gastrointestinal cancer, 2 patients with renal cancer, and 1 patient with multiple myeloma. The age was 59.48 ± 6.88 years. They underwent 28 kyphoplasty operations, including 18 thoracic and 10 lumbar. General characteristics of the patients are summarized in Table 1. Case A: Imaging data for a typical patient with L_1_ and L_2_ metastatic spinal tumors without spinal cord compression with posterior wall destruction is shown in Figs 4, 5, and 6. Case B: Another typical patient with T_5_ metastatic spinal tumors is shown in Figs 7 and 8.

**Table 1:** General characteristics of the patients

Characteristic	Value
Patients	
Number	23
Age (years)	59.48 ± 6.88
Gender (F/M)	13/10
Primary tumors	
Breast	9
Lung	7
Liver	2
Gastrointestinal	2
Kidney	2
Multiple myeloma	1
Site of treated vertebrae	
Thoracic	18
Lumbar	10
Surgery	
Operative time (min)	162.61 ± 33.47
Blood loss (mL)	230.87 ± 93.76
Time from surgery to discharge (day)	4.35 ± 2.42
Cement volume (mL)	3.67 ± 0.63
Complication	
Cement leakage	0

### *Surgical Operation*

According to statistics, the operation time of these patients was 162.61 ± 33.47 min, the amount of bleeding was 230.87 ± 93.76 mL, the time from operation to discharge was 4.35 ± 2.42 days, and the volume of bone cement was 3.67 ± 0.63 mL. No cement leakage occurred in any of the patients postoperatively.

### *Clinical and Radiographic Evaluation*

#### *Vertebral Height*

The results of clinical and radiological assessments are shown in Table 2. The height of the middle vertebral body increased from 14.47 ± 2.96 mm before the operation to 20.18 ± 2.94 mm (*P* = 0.000), and remained at 20.44 to 3.01 mm 1 year after the operation. The height of the posterior vertebral body increased from 16.56 ± 3.07 mm before the operation to 22.79 ± 4.00 mm (*P* = 0.000), and 22.45 ± 3.88 mm 1 year after the operation.

**Table 2:** Radiographic and clinical evaluation

Evaluation	Value	*P*‐value
Visual analogue scale		
Preoperative	7.04 ± 1.07	
Postoperative	2.65 ± 0.93	0.000
1 year postoperatively	2.57 ± 0.79	0.000
SF‐36, BP		
Preoperative	16.30 ± 6.23	
Postoperative	54.22 ± 11.75	0.000
1 year postoperatively	54.70 ± 9.31	0.000
SF‐36, PF		
Preoperative	25.65 ± 7.58	
Postoperative	51.09 ± 7.38	0.000
1 year postoperatively	52.39 ± 7.52	0.000
SF‐36, VT		
Preoperative	29.78 ± 5.53	
Postoperative	54.13 ± 4.92	0.000
1 year postoperatively	55.65 ± 11.80	0.000
SF‐36, SF		
Preoperative	27.17 ± 11.09	
Postoperative	54.35 ± 13.39	0.000
1 year postoperatively	53.80 ± 10.95	0.000
Middle vertebral height		
Preoperative	14.47 ± 2.96	
Postoperative	20.18 ± 2.94	0.000
1 year postoperatively	20.44 ± 3.01	0.000
Posterior vertebral height		
Preoperative	16.56 ± 3.07	
Postoperative	22.79 ± 4.00	0.000
1 year postoperatively	22.45 ± 3.88	0.000

#### *Visual analogue scale*

The VAS score decreased from 7.04 ± 1.07 to 2.65 ± 0.93 before the operation (*P* = 0.000), and remained at 2.57 ± 0.79 1 year after the operation.

#### *SF‐36*

The SF‐36 scores for BP increased from 16.30 ± 6.23 to 54.22 ± 11.75 before the operation (*P* = 0.000) and remained at 54.70 ± 9.31 1 year after the operation. The SF‐36 scores for PF increased from 25.65 ± 7.58 to 51.09 ± 7.38 before the operation (*P* = 0.000) and remained at 52.39 ± 7.52 1 year after the operation. The SF‐36 scores for VT increased from 29.78 ± 5.53 to 54.13 ± 4.92 before the operation (*P* = 0.000) and remained at 55.65 ± 11.80 1 year after the operation. The SF‐36 scores for SF increased from 27.17 ± 11.09 to 54.35 ± 13.39 before the operation (*P* = 0.000) and remained at 53.80 ± 10.95 1 year after the operation.

#### *Pedicle Screw Position*

In the study, a total of 92 pedicle screws were evaluated and 85 screws were Grade A. The accuracy of pedicle screws was 92.3% (85/92), and only 7 pedicle screws broke through the pedicle and were evaluated as Grade B.

## Discussion

Harrington proposed a classification scheme for metastatic spinal tumors according to the extent of bone and nerve invasion by tumor tissues^15^: (i) no neurological symptoms; (ii) the spinal column was invaded, but there was no collapse or instability; (iii) neurological dysfunction (sensory or motor), with no bone involvement; (iv) vertebral body collapse, and pain from mechanical causes or instability, but no significant nerve injury; and (v) vertebral collapse or instability and severe nerve injury. Patients in categories 1, 2, and 3 can be treated with non‐operative methods such as radiotherapy and chemotherapy, while patients in categories 4 and 5 need surgical intervention. However, for non‐surgical patients, there is a high risk that the diseased vertebrae will eventually develop pathological fractures^16^. Tokuhashi *et al*. put forward a preoperative scoring system for patients with metastatic spinal tumors from six aspects^17^: medical status, extraspinal metastasis, vertebral metastasis, visceral metastasis, primary tumor types, and neurological dysfunction. Then, the corresponding treatment methods were selected according to the scoring. Complete resection appears to be the perfect strategy for patients with spinal tumors. However, secondary spinal tumors are far away from the primary lesion, and complete resection of spinal lesions can cause massive blood loss and other complications, and even reduce the expected survival time of patients^18^. Moreover, the complication and mortality rates as a result of long operation times, massive blood loss, extensive soft tissue dissection, and long‐term hospitalization are still quite high^19^. No studies have shown that complete resection can improve quality of life and survival rates. Some open partial excisions, such as fragmentary excision and eggshell scraping, still result in blood loss, long‐term hospitalization, and vertebral instability, even after pedicle screw fixation.

O‐arm CT navigation technology provides high‐quality two‐dimensional and three‐dimensional real‐time intraoperative images and ensures the accuracy of high‐difficulty operations. O‐arm CT navigation technology mainly solves three problems: first, during the CT scan, the doctor can temporarily separate from the radiation source; second, it can reduce the number of radiation treatments and the radiation dose during the operation, so that the safety of doctors and patients can be improved; and, third, combining with the navigation technology, the operation can achieve accurate surgery in real‐time 3D space, with minimally invasive surgery.

In most cases, palliative surgery is used for metastatic spinal tumors. Surgery is conducive to removing the lesion for biopsy to further clarify the diagnosis and determine the next treatment plan, which can indirectly prolong the life span. In this study, metastatic lesions of the spine were first found in the 10 patients, and PET‐CT could identify the primary lesions. Based on the biopsy of the lesions, further definite pathological diagnosis is made and cancer treatment is carried out. In our study, we performed the operation for 162.61 ± 33.47 min. The blood loss during the operation was 230.87 ± 93.76 mL and the time from operation to discharge was 4.35 ± 2.42 days. The above three parameters are significantly lower than those of open surgery, and even lower than those of minimally invasive surgery reported in some cases^20^, ^21^. The main reason may be that the O‐arm navigation system provides a clearer field of vision, making the operation minimally invasive, safe, and accurate^22^. Therefore, compared with traditional open surgery, minimally invasive surgery, such as minimally invasive pedicle screw fixation combined with percutaneous kyphoplasty, has great advantages in regard to operation time, soft tissue dissection, blood loss, and hospital stay, which are important factors affecting expected survival^23^.

The main symptom of patients with metastatic spinal disease is pain, which is also the first problem to be solved in palliative treatment. Mourbauer *et al*. published the first description of minimally invasive spine surgery for metastatic spinal diseases, in which patients showed significant relief in pain and neurological improvement after surgery^24^. Harrington first implemented bone cement augmentation to relieve pain from metastatic spinal diseases^25^. At present, the two most popular methods of bone cement enhancement are vertebroplasty and kyphoplasty. The mechanism of pain relief is still controversial. Restoring spinal stability may be the main reason for pain relief. In addition, the toxicity of bone cement monomer and exothermic aggregation may lead to necrosis of pain receptors in bone. Kostuik *et al*. believed that surgical intervention depended on the stability of the spine^26^. They attempted to define stability in terms of the concept of two columns of the spine. The whole vertebral body consists of the anterior column, while the pedicle, lamina and spinous process are classified as the posterior column. The anterior and posterior columns are further divided into six regions. If no more than two regions are destroyed, the spine is stable, but once three or more regions are destroyed, the spine is unstable. Denis *et al*. define the three‐column theory of the spine, which indicates instability if it conforms to any of the following characteristics: deformity, vertebral collapse greater than 50%, involvement of the three columns, or involvement of the same column in two or more adjacent levels^27^. Therefore, reconstruction of spinal stability is necessary for metastatic spinal tumors.

In our study, all patients suffered from severe pain and structural damage to the spine due to tumor invasion, with no spinal cord compression occurring. Cement augmentation can only restore the height of the anterior and middle columns of the diseased vertebra, while the spine remains unstable due to the lack of support on the posterior column, and the defect of the vertebral wall increases the risk of cement leakage^28^. Although pedicle screw fixation can restore the height and physiological curvature of the vertebral body, the anterior column support is insufficient, and the strength and height of the diseased vertebral body cannot be restored. Moreover, in elderly patients, bone destruction may lead to loosening and fracture of the screw after operation^29^. Therefore, the combined operation can not only restore the height of the vertebral body but also strengthen the diseased vertebral body, reduce the stress of the screw, maintain the height of the vertebral body after the operation, and improve the success rate of pedicle screw fixation.

Cement leakage is the most common complication of kyphoplasty; the factors affecting cement leakage include cement viscosity, cement volume, and integrity of the vertebral wall^30^. Therefore, patients with metastatic spinal tumors with posterior wall damage are more likely to have cement leakage during kyphoplasty surgery. In theory, complete posterior longitudinal ligament and anterior longitudinal ligament can prevent cement leakage from the vertebral wall. Yang *et al*. discussed kyphoplasty in detail, as well as a variety of bone cement injection techniques^31^, such as a graded infusion technique and a temperature‐increasing cement delivery system. Good injection technology can reduce or even avoid the leakage of bone cement.

Even though studies show that surgery is an important part of the treatment of metastatic spinal tumors, preoperative and/or postoperative radiotherapy is still essential^32^. The timing of radiotherapy should be 1–2 weeks before and after surgery to avoid postoperative complications. The main goal of radiotherapy is to reduce the pain caused by metastasis to achieve local control of the lesion. However, it is ineffective in preventing the collapse of pathological vertebral fractures in a vertebral body with bone destruction^33^. Many studies have shown that the treatment of malignant tumors requires multidisciplinary cooperation. In recent years, there has been a lack of systematic integration of new technologies, such as minimally invasive surgery, targeted therapy and immunotherapy, so the expected survival and the quality of life of patients with advanced tumors are not maximized^34^.

In this study, the biggest limitation is that we only elaborate on the short‐term efficacy of minimally invasive treatment and the lack of long‐term observation combined with other treatments. In addition, because spinal cord decompression is involved, we excluded patients with spinal cord compression. Furthermore, we only recorded the time from surgery to discharge, and not the time from surgery to the commencement of antitumor treatment. Finally, the study is retrospective.

### *Conclusion*

In this series, minimally invasive pedicle screw fixation combined with percutaneous kyphoplasty is safe and effective in the treatment of metastatic spinal tumors with posterior wall destruction in the short term. Postoperative patients can quickly recover and transfer to other departments and undergo follow‐up anti‐cancer treatment.
